# An Evaluation of *Spodoptera littoralis* and *Spodoptera exigua* as Natural Prey for the Generalist Predator *Chrysoperla carnea*

**DOI:** 10.3390/insects16020167

**Published:** 2025-02-05

**Authors:** Agustín Garzón, Óscar Giovanni Gutiérrez-Cárdenas, Beatriz Dáder, Pilar Medina, Ángeles Adán

**Affiliations:** 1 Escuela Técnica Superior de Ingeniería Agronómica, Alimentaria y de Biosistemas, Universidad Politécnica de Madrid, Avenida Puerta de Hierro 2, 28040 Madrid, Spain; oggutierrez25@gmail.com (Ó.G.G.-C.); beatriz.dader@upm.es (B.D.); pilar.medina@upm.es (P.M.); angeles.adan@upm.es (Á.A.); 2 Genómica Alimentaria, Universidad de La Ciénega del Estado de Michoacán de Ocampo, Sahuayo 59103, Michoacán, Mexico

**Keywords:** chrysopid, generalist predator, noctuids, natural prey, predator preference, predator development

## Abstract

*Chrysoperla carnea* (Stephens) is a common generalist predator in agroecosystems, frequently used for the control of soft-bodied pests in augmentative releases. Better knowledge of its interactions with secondary natural prey is necessary to optimize field biocontrol performance. This work evaluates the eggs and early larvae of *Spodoptera exigua* (Hübner) and *Spodoptera littoralis* (Boisduval) as natural prey for the third larval instar of *C. carnea*, and the predator preferences for different prey stages under varying conditions, assessing the impact on its life cycle and reproduction, in comparison with commercial diets based on *Ephestia kuehniella* Zeller eggs. Prey choice results were uniform for each dual prey combination irrespective of previous predator conditions (larvae number or fasting). *Chrysoperla carnea* did not show preference for *Ephestia* or *Spodoptera* eggs, but preferred eggs prior to larvae of the natural prey. Diets based on the frozen eggs of natural and factitious prey species showed similar effects on the development and reproduction of predator, but when *C. carnea* fed on *S. exigua* larvae, several negative effects were assessed, such as longer preimaginal development, lower adult emergence, and a reduced fecundity. These results are helpful for improving the use of lacewings as biocontrol agents in pest control programs.

## Simple Summary

The role of generalist predators in the biological control of crop pests is increasingly recognized due to their resilience and adaptability. Understanding their feeding habits is crucial for their conservation in agroecosystems and for assessing their impact on various pests. *Chrysoperla carnea*, a significant biological control agent, is known for its voracious predation. Despite extensive knowledge about its predation on aphids, there is limited research on its interactions with other natural prey. This study focuses on evaluating the eggs and early larvae of the beet armyworm (*Spodoptera exigua*) and the cotton leafworm (*Spodoptera littoralis*) as potential natural prey for *C. carnea*. Interaction and fasting enhanced prey encounters among lacewing larvae. Mobile larvae in the dual combination of preys reduced no-choice instances. When two chrysopid larvae were present, they did not attack each other, indicating increased chances of predator–prey encounters. *Chrysoperla carnea* larvae adapted to preying on *S. littoralis* eggs, but discriminated against its second-instar larvae. *Spodoptera littoralis* eggs showed optimal nutritional quality. Diets based on live *S. exigua* larvae negatively impacted predator development and fecundity, while moth eggs improved adult reserves. These findings contribute to the optimization of lacewing use in pest control programs.

## 1. Introduction

Nowadays, the importance of generalist predators in the biological control of crop pests is well established because they are resilient and adapted to temporary periods of low target pest densities [1,2]. However, knowing more about their broad feeding habits will be useful for their conservation in the agroecosystem and to measure impacts on different pests [3]. The dynamic relationship between a generalist predator and a target pest is affected by the availability of other non-target food resources, by prey choice, and by the degree of polyphagy [1].

*Chrysoperla carnea* (Stephens) (Neuroptera: Chrysopidae) is an important biological control agent, both as natural predator and for augmentative releases [4,5,6,7]. Chrysopid larvae are highly voracious predators, capable of consuming their own body weight in prey on a daily basis, irrespective of the larval instar [8]. Although considered a homopterophilic predator (main activity focused on aphids) [9], it is also an opportunistic generalist in the selection of prey. The potential range of prey includes many soft-bodied insects such as other Hemiptera, thrips, mites, and the eggs and larvae of Lepidoptera, Coleoptera, and Diptera [10,11,12].

Other notable characteristics for its auxiliary role are a wide distribution and broad tolerance to different ecological factors and, therefore, a great adaptability under field conditions compared to other predators [6,13,14,15]. In addition, it is commercially available thanks to mass-rearing with the frozen or irradiated eggs of factitious prey species such as *Sitotroga* sp. (Lepidoptera: Gelechiidae), *Ephestia* sp., or *Corcyra* sp. (Lepidoptera: Pyralidae) [6,11].

Most of the knowledge about the predator activity of *C. carnea* is focused on aphids, its target pest in augmentative releases [16,17], whereas few studies have been conducted on other natural prey species. For example, it is the main natural enemy of the carpophagous generation of *Prays oleae* (Bernard) (Lepidoptera: Praydidae) at olive orchards in Spain and Portugal [7,14]. A better understanding of the feeding habits and performance of *C. carnea* when consuming different natural prey species would contribute to the optimization of augmentative releases and the conservation of this biocontrol agent.

This work evaluates the suitability of eggs and early larvae of the beet armyworm *Spodoptera exigua* (Hübner) and the cotton leafworm *Spodoptera littoralis* (Boisduval) (Lepidoptera: Noctuidae) as natural prey for the third larval instar of *C. carnea*. Both noctuid species are considered important pests of protected crops in Southeastern Spain [18], but they cause damage to many crops because of their polyphagy [19]. *Spodoptera exigua* is a cosmopolitan species that can be found across many countries in the European and Mediterranean Plant Protection Organization (EPPO) region. It targets vegetables, cotton, and ornamental plants, affecting both outdoor and protected environments. In the greenhouses of Southeastern Spain, it is a major pest for tomato (*Solanum lycopersicum* L.) and sweet pepper (*Capsicum annuum* L.) [18,20,21,22]. Meanwhile, *S. littoralis*, listed on the EPPO A2 quarantine list [23], inhabits the Palearctic region and can damage over 40 plant families, comprising approximately 87 economically important species [19,24,25].

In our study, two different objectives were assessed. First, we examined predator preference for the eggs of natural and factitious prey and for two stages (egg and larvae) of the natural prey in different conditions of predator fasting and number of individuals. Second, we studied the performance of diets based on *Spodoptera* eggs or larvae compared with commercial diets based on *Ephestia kuehniella* Zeller (Lepidoptera: Pyralidae) eggs.

## 2. Materials and Methods

### 2.1. Biological Material

*Chrysoperla carnea* was delivered in 500 mL containers with 500 L2 larvae each and buckwheat hulls as carrier (Insectaria, Logroño, Spain). Once received, larvae were individualized in ventilated 24-cell multi-well plates with *E. kuehniella* eggs ad libitum (Insectaria, Logroño, Spain; EPHEScontrol^®^, Agrobio, Almería, Spain).

*Spodoptera littoralis* was collected on *Medicago sativa* (L.) in Los Palacios (Sevilla, Spain). *Spodoptera exigua* belonged to a laboratory population initially collected in Murcia (Spain). Both noctuids were continuously reared on a semi-synthetic diet [26]. The three insect species were maintained inside a walk-in chamber (4.25 × 2 × 2.5 m) with controlled conditions (16:8 L:D photoperiod, 24.5 ± 1.8 °C temperature and 56.1 ± 10.2% relative humidity).

### 2.2. Spodoptera littoralis Eggs as Natural Prey for Chrysoperla carnea L3 Larvae

#### 2.2.1. Dual Choice: *Ephestia kuehniella* vs. *Spodoptera littoralis* Eggs

We tested the preference of *C. carnea* for *S. littoralis* or *E. kuehniella* eggs (<24 h old). *Spodoptera littoralis* eggs were offered as they were laid on the original substrate for female oviposition (filter paper), but were previously frozen to avoid hatching and have the same nutritional conditions as *E. kuehniella* eggs (commercial diet), which were purchased frozen. Patches of eggs (20 mg) were fixed on 2 × 2 cm pieces of filter paper. The influence of the feeding condition (with the predator fasted during the last 24 h or fed ad libitum) and the influence of number (one or two newly molted *C. carnea* L3 larvae) were evaluated in their four combinations. Eggs were placed inside a Petri dish (9 cm), equidistant from the central position in which the predator(s) were released. The maximum visual observation time to register the first prey choice was established in 30 min. Otherwise, the repetition was considered as no choice. A predator was considered to have made a choice when it fed consistently on the prey for one minute. The time that elapsed until a choice was made was recorded. When two *C. carnea* were tested together, only the first choice was considered. Moreover, the relative position between them was registered (close, same half of the dish; or distant, different half of the dish). A randomized set of replicates from each combination were performed every day until the completion of the assay (n = 58 replicates per each combination predator number/feeding status).

#### 2.2.2. *Spodoptera littoralis* Eggs as Diet for *Chrysoperla carnea* L3 Larvae

We compared the effect of feeding *C. carnea* with different prey eggs in the development and reproduction of the predator. Chrysopid L3 larvae (2–3 mg weight) were held individually inside ventilated plastic cages (3.3 cm diameter) containing filter paper as shelter substrate and prey eggs. Two prey species were studied, either frozen *S. littoralis* or *E. kuehniella* eggs (n = 22 replicates per treatment). Food was daily renewed (20 ± 0.1 mg per predator larva). Once *C. carnea* larvae pupated, they were grouped by treatment inside a ventilated plastic cage (50 × 35 × 35 cm) containing adult food [27] and water. Once adults emerged, pairings of one male and one female were placed inside a ventilated plastic cage (12 cm diameter, 5 cm high) containing food and water (n = 6–7 replicates per treatment). A gauze was placed on top of the cage as an oviposition substrate. The gauze was changed every two days, and the number of eggs was recorded. The parameters evaluated were pupae formation (proportion of pupae/larvae), pupae weight (mg, measured the second day after pupation), adult emergence (proportion of adults/larvae), and fecundity (eggs/day, during 10 days after the observation of the first oviposition). Cages with insects were placed in a randomized disposition inside the walk-in chamber to avoid a positional effect.

### 2.3. Spodoptera sp. Larvae as Prey for Chrysoperla carnea L3 Larvae

#### 2.3.1. Dual Choice: *Spodoptera littoralis* Eggs vs. L2 Larvae

The preference of *C. carnea* L3 larvae for *S. littoralis* eggs or L2 larvae was studied under the same conditions described in Section 2.2.1 (n = 58 replicates per each combination predator number/feeding status). Patches of eggs (20 mg) were fixed onto pieces of filter paper (2 × 2 cm). Noctuid larvae were directly taken from the general rearing, choosing individuals with good mobility and 3 mm smaller than chrysopid larvae. Eggs and one larva were placed inside a Petri dish (9 cm diameter), equidistant from the central position used as the releasing point for the predator(s). Then, the procedure described in Section 2.2.1 was followed.

#### 2.3.2. *Spodoptera exigua* L2 Larvae as Diet for *Chrysoperla carnea* L3 Larvae

We studied the effects of feeding *C. carnea* with *S. exigua* L2 larvae on the performance of the predator. As *C. carnea* did not choose *S. littoralis* larvae in the dual choice assay (Section 2.3.1), the cotton leafworm was replaced by the beet armyworm due to its smaller size. In addition, two commercial diets with different proportions of the factitious prey were evaluated, frozen *E. kuehniella* eggs and a mixture of *E. kuehniella* eggs and cysts of the crustacean *Artemia* spp. (1:5 *w*/*w*) (Entofood^®^, Koppert, Almería, Spain). Before starting the assay, two different populations of the predator were reared for two generations with these diets. In the third generation, four groups (two from each population) of neonate larvae were held individually inside disposable blisters (3.1 × 1.5 × 2.5 cm per alveoli) (Arapack, Zaragoza, Spain) and reared with the corresponding aforementioned diet until L3 molting. At that moment, the diet of one group from each population was replaced by live *S. exigua* L2 larvae, whereas the other group kept the initial diet. Thus, four treatments were considered: (1) *E. kuehniella* (E), (2) *E. kuehniella* + *Artemia* spp. (EA), (3) *E. kuehniella* + *S. exigua* (ES), and (4) *E. kuehniella* + *Artemia* spp. + *S. exigua* (EAS) (n = 70 replicates per treatment). Food was daily renewed (200 mg commercial diet or 10 *S. exigua* larvae). The number of larvae consumed per day had been previously determined to be 8.7 ± 0.1, therefore a slightly higher number was offered [28]. Once adults emerged, groups of three males and three females were placed together inside a ventilated plastic cage (19.5 × 16.5 × 8 cm) containing food and water (n = 14 replicates per treatment) [15]. A gauze was placed on top of the cage as an oviposition substrate. The gauze was changed every two days and the number of eggs was recorded. The parameters evaluated were: L3 developmental time (days), pupae formation (proportion of pupae/larvae), pupae weight (mg, random sample of 35 pupae per treatment measured on the second day after pupation), developmental time of pupae (days), adult emergence (proportion of adults/larvae), fecundity (eggs/female and day, during 14 days after the observation of the first oviposition), fertility (% neonate larvae/eggs, gauzes retrieved on days 4, 7, 11, and 14), and adult survival (% live adults/total adults, 30 days after emergence). We evaluated more parameters than in the feeding bioassay of Section 2.2 because the nutritional quality of the larvae has been less studied than diets based on eggs. Cages with insects were placed in a randomized disposition inside the walk-in chamber to avoid a positional effect.

### 2.4. Statistics

All data were analyzed using SPSS Statistics Software Package for Windows version 24.0.0.0 (SPSS Inc., Chicago, IL, USA). In the dual choice assays, the results of the first choice were analyzed with a Chi-square (*χ*^2^) (one-sample test). The time to first choice was compared with Student’s *t*-test (*p* < 0.05). Parameters from the feeding assays were analyzed by Student’s *t*-test (*p* < 0.05) or unifactorial ANOVA followed by an LSD test (*p* < 0.05). The categorical data of pupation and emergence were assessed with a 2 × 2 Chi-square (*χ*^2^) contingency table.

## 3. Results

### 3.1. Spodoptera littoralis Eggs as Natural Prey for Chrysoperla carnea L3 Larvae

#### 3.1.1. Dual Choice: *Ephestia kuehniella* vs. *Spodoptera littoralis* Eggs

**Figure 1:**
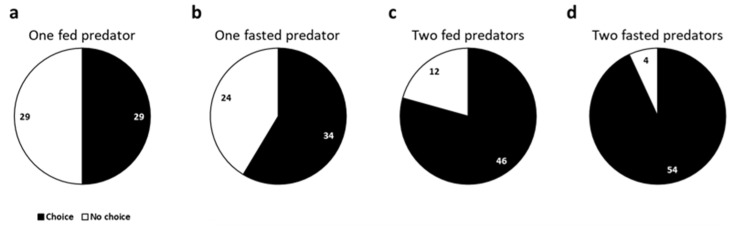
The proportion of total observations (choice and no choice) of *Chrysoperla carnea* L3 larvae to the offering of *Spodoptera littoralis* or *Ephestia kuehniella* eggs under different conditions: (**a**) one fed predator; (**b**) one fasted predator; (**c**) two fed predators; (**d**) two fasted predators. Responses within 30 min are represented in black, and no responses in white. The numbers represent the observations for every option.

Figure 1 only shows the proportion of choice and no choice data by *C. carnea* L3 larvae of the total observations (which means whether there was a response or not), regardless of preference. The presence of two predator larvae in the same arena significantly decreased the instances of no choice regardless of the feeding status of the larvae (fasted: *χ*^2^ = 43.10, *p* < 0.001; fed: *χ*^2^ = 19.93, *p* < 0.001) in the 30 min observation time (Figure 1c,d). There were no statistical differences between choice and no choice data with a single predator larva (fasted: *χ*^2^ = 1.72, *p* = 0.189; fed: *χ*^2^ = 0, *p* = 1) (Figure 1a,b). We did not observe a significant pattern in the relative position between the two *C. carnea* larvae within the arena (close or distant) when they competed for the same prey. In addition, no intraspecific fighting and predation events were observed.

**Figure 2:**
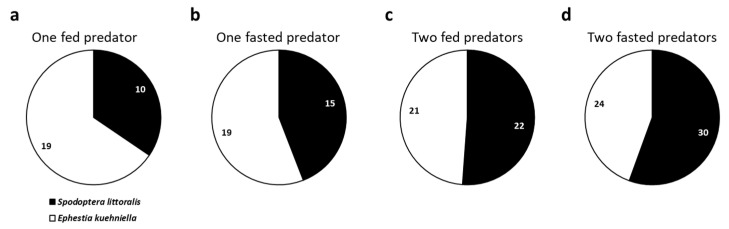
The proportion of preference of *Chrysoperla carnea* L3 larvae for *Spodoptera littoralis* or *Ephestia kuehniella* eggs under different conditions: (**a**) one fed predator; (**b**) one fasted predator; (**c**) two fed predators; (**d**) two fasted predators. A preference for *S. littoralis* is represented in black, and a preference for *E. kuehniella* in white. The numbers represent the observations for every option.

Despite being traditionally fed with *E. kuehniella* for mass-rearing, *C. carnea* did not show a preference for this host egg in any condition tested of larvae number and feeding status (one fed larva: *χ*^2^ *=* 2.79, *p* = 0.095; one fasted larva: *χ*^2^ = 0.47, *p* = 0.493; two fed larvae: *χ*^2^ *=* 0.02, *p* = 0.879; two fasted larvae: *χ*^2^ *=* 0.67, *p* = 0.414) (Figure 2a–d). In the case of two fed predators, no conclusions were possible in 3 out of 46 observations, so these were not included in the analysis (Figure 1c and Figure 2c).

The response time of the first choice was only significantly different when two *C. carnea* larva competed in conditions of starvation and chose *E. kuehniella* (Table 1). The response time, regardless of the choice that *C. carnea* made, was also compared to discriminate if predator conditions (number and feeding status) influenced their response. No statistical differences could be found.

#### 3.1.2. *Spodoptera littoralis* Eggs as Diet for *Chrysoperla carnea* L3 Larvae

After feeding *C. carnea* L3 larvae with either *S. littoralis* or *E. kuehniella* eggs until adulthood, the proportion of larvae that reached pupal stage was similar between diets, but the pupae weight was significantly higher when fed on *S. littoralis* (Table 2). A significantly higher number of larvae that fed on *S. littoralis* reached adult stage. The fecundity was not statistically different between the diets (Table 2).

### 3.2. Spodoptera sp. Larvae as Prey for Chrysoperla carnea L3 Larvae

#### 3.2.1. Dual Choice: *Spodoptera littoralis* Eggs vs. L2 Larvae

**Figure 3:**
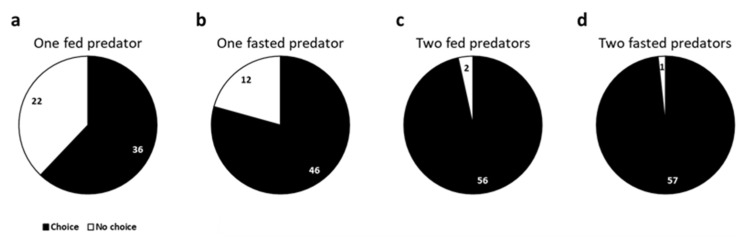
The proportion of total observations (choice and no choice) of *Chrysoperla carnea* L3 larvae regarding the offering of *Spodoptera littoralis* eggs or L2 larvae under different conditions: (**a**) one fed predator; (**b**) one fasted predator; (**c**) two fed predators; (**d**) two fasted predators. Responses within 30 min are represented in black, and no responses in white. The numbers represent the observations for every option.

In this situation, the number of replicates with a choice made (regardless of the prey selected) increased in all cases compared with the dual choice of *S. littoralis* vs. *E. kuehniella* eggs. Choice responses were significantly higher than no choice with two predator larvae and fasting (one fasted larva: *χ*^2^ = 19.93, *p* < 0.001; two fasted larvae: *χ*^2^ = 54.07, *p* < 0.001; two fed larvae: *χ*^2^ = 50.28, *p* < 0.001) (Figure 3b–d). Furthermore, when two fasted *C. carnea* larvae competed for the same prey, they coincided in the same half of the arena (*χ*^2^ = 5.79, *p* = 0.02;) but no fight was observed between them.

**Figure 4:**
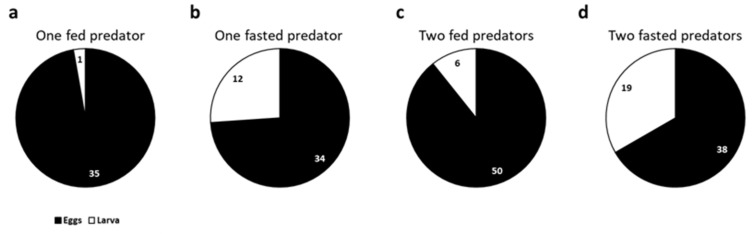
The proportion of preference of *Chrysoperla carnea* L3 larvae for *Spodoptera littoralis* eggs or larva under different conditions: (**a**) one fed predator; (**b**) one fasted predator; (**c**) two fed predators; (**d**) two fasted predators. A preference for eggs is represented in black, and a preference for larva in white. The numbers represent the observations for every option.

The predator clearly preferred *S. littoralis* eggs to larvae in every condition tested (one fed larva: *χ*^2^ = 32.11, *p* < 0.001; one fasted larva: *χ*^2^ = 10.52, *p* = 0.001; two fed larvae: *χ*^2^ = 34.57, *p* < 0.001; two fasted larvae: *χ*^2^ = 6.33, *p* = 0.012) (Figure 4). Aside from this significant preference, a higher number of larvae was preyed on if the predators had been starved (Figure 4b,d).

No statistical differences regarding the response time of the first choice between eggs or larva were found (Table 3). Not taking into account the choice that *C. carnea* made, the response time of two fed larvae was significantly lower compared to one fasted larva (*F*_3,191_ = 3.26; *p* = 0.02).

#### 3.2.2. *Spodoptera exigua* L2 Larvae as Diet for *Chrysoperla carnea* L3 Larvae

The duration of the chrysopid L3 larval and pupae stages was significantly longer when they fed on live *S. exigua* larvae (ES, EAS) after L3 molting (Table 4). Chrysopid L3 larvae fed with diets containing *S. exigua* pupated significantly less well. Also, the weight of these pupae was significantly lower than those fed with *E. kuehniella*. In line with the observation of the pupae, the emergence of adults previously fed with *S. exigua* was significantly poorer.

The fecundity of chrysopid females was significantly lower if they had fed on diets containing live *S. exigua* larvae (ES, EAS) (Table 5). Finally, there were no significant differences among diets on *C. carnea* fertility and adult survival after 30 days.

## 4. Discussion

The eggs and early larvae of *Spodoptera* are two different prey targets for the natural enemy *C. carnea*. The results of the choice trials enable us to propose several hypotheses regarding the behavior of the last and most voracious instar of the predator toward these pests. Predator–larvae interaction and, to a lesser extent, fasting increased encounters with prey (more choices reported). Additionally, the presence of mobile larvae in the dual-prey combination significantly reduced the number of ‘no choice’ responses, nearly halving it compared to the other prey combination (eggs from two species). Hungry larvae initially increase their activity to find prey, but decreased it later if no prey was encountered [9]. Most green lacewing larvae carry out an active and random search for prey until they meet it, and only within a short distance they appear to be positively stimulated by different factors, such as honeydew and lepidopteran scales [9], or the mobility of preys [29]. In our study, when two lacewing larvae were present in the arena, we did not observe any attacks between them, though they also did not distance themselves from each other. Therefore, the higher number of choices involving two lacewing larvae likely reflects an increased probability of a chance predator–prey encounter. Interestingly, none of these factors (larvae number or fasting) affected the prey selection results, as the outcomes remained consistent for each dual prey combination.

A rearing diet based in factitious prey may affect the behavioral response of entomophagous compared to natural prey, because the artificial conditions can affect the quality of mass-reared predators [30]. In the case of *C. carnea*, larvae could easily switch to preying on the eggs of the natural prey *S. littoralis*, even if they had previously been fed on *E. kuehniella* eggs for generations. On the contrary, when the dual combination was immature stages of the natural prey, the predator clearly discriminated against second-instar larvae of *S. littoralis*, suggesting that under field conditions, the attack on the pest larvae would be of little importance. Meier and Hilbeck [31] found that the late larval instar of *C. carnea* prefers to prey on groups of 40 aphids rather than 40 neonate *S. littoralis*. Instead, other studies report a preference for young lepidopteran larvae over eggs [5,29] or aphids [28]. Prey identification in lacewing larvae is conditioned by contact with the palpi and/or the antennae and probing [9]. However, in most cases, there was no contact with *S. littoralis* larvae before choosing the eggs. Considering that lepidopteran larvae can defend themselves with their mandibles and cause injuries to lacewings despite their smaller size [28,32], and can also escape, the risk and effort of hunting are probably the main factors that determine the choice of one prey or another.

When comparing the two diets based on the frozen eggs of natural and factitious prey species, *S. littoralis* eggs showed similar or even higher nutritional quality than *E. kuehniella* eggs for the third instar of predator larvae. Otherwise, Braghini et al. [33] reported a faster larval development and better reproductive parameters when *Chrysoperla externa* (Hagen) (Neuroptera: Chrysopidae) consumed *E. kuehniella* eggs compared with the eggs of *Diatraea saccharalis* (F) (Lepidoptera: Crambidae).

A broader evaluation of the diet based on live *S. exigua* larvae showed a general negative impact on the predator’s performance compared with diets based on *E. kuehniella* eggs (with or without *Artemia* sp. cysts). Lacewings larvae confined with the live prey, and without choice, showed a high consumption rate of *S. exigua* larvae. Despite this, the preimaginal development and preoviposition days of *C. carnea* were longer compared with the egg diets, suggesting a lower nutritional quality [34]. *Chrysoperla carnea* also shows faster development when they prey on *Mamestra brassicae* (L) (Lepidoptera: Noctuidae) eggs compared to neonate larvae [35]. Considering that *C. carnea* is a sucking predator, neither eggshell nor the exoskeleton are consumed, and the larval exoskeleton represents a higher proportion per unit weight than the eggshell [35]. Eggs appear to be more nutritious than larvae but also, lacewings expend more energy in handling mobile prey, contributing to slower development [5]. In addition, the delay observed during the third larval instar may be partly associated with the subsequent mortality increase during metamorphosis.

We noticed a decrease in adult emergence, in accordance with *C. carnea* fed on *S. littoralis* larvae compared to diets based on *Rhopalosiphum padi* (L) (Homoptera: Aphididae) or *Tetranychus urticae* (Koch) (Acari: Tetranychidae) [32]. Although the longevity of the predator adults was not affected by the larvae diet, fecundity was severely reduced. In line with our results, the fecundity of *Mallada boninensis* (Okamoto) (Neuroptera: Chrysopidae) is reduced by half when it feeds on neonate noctuids compared to *Corcyra cephalonica* (Stainton) (Lepidoptera: Pyralidae) eggs [36]. Therefore, diets based on moth eggs may allow a higher accumulation of reserves, resulting in the higher fecundity of adults. Predators can maintain survivorship but not reproduction if feeding is deficient [37].

Regarding the comparison between the two diets of *E. kuehniella* eggs, there were differences only in pupae weight. The highest weight registered when the predator exclusively fed on *E. kuehniella* eggs coincides with past records [38,39]. Adding *Artemia* spp. to the diet reduced pupae weight. The cocoon silk has an amino acid composition mainly based on non-essentials (glycine, alanine, serine) [40]. The richer content of glycine and alanine of *E. kuehniella* eggs compared to *Artemia* spp. cysts may explain our results [41]. The formation of lighter cocoons was also associated with a shorter pupal development.

To sum up, the eggs and larvae of *S. littoralis* and *S. exigua* are natural prey that *C. carnea* can find on different crops. This research on the preferences and performance of lacewing larvae regarding these prey species contributes to a better understanding of its nutritional ecology and can help optimize their use in pest control programs. However, further studies are required to evaluate the field adaptation of this generalist predator in a more realistic environment that includes a greater prey diversity.

## Figures and Tables

**Table 1:** The response time (minutes) of the first choice of *Chrysoperla carnea* L3 larvae under different conditions of predator number (one or two larvae) and feeding status (fed or fasted in the previous 24 h). The letters stand for significant differences between treatments by Student’s *t*-test (*p* < 0.05).

Feeding Status	Fasted		Fed	
Larvae Number	One Larva	Two Larvae	One Larva	Two Larvae
*S. littoralis*	7.42 ± 2.04	10.76 ± 2.83 b	2.95 ± 0.79	4.41 ± 1.40
*E. kuehniella*	6.88 ± 1.67	4.65 ± 0.88 a	5.39 ± 1.55	3.56 ± 0.86
Statistics	*t* = −0.20; *p* = 0.84	*t* = −2.59; *p* = 0.02	*t* = 1.48; *p* = 0.14	*t* = −0.51; *p* = 0.61

**Table 2:** Comparison of the development of *Chrysoperla carnea* L3 larvae after feeding on *Spodoptera littoralis* or *Ephestia kuehniella* eggs. Letters stand for significant differences between treatments by Student’s *t*-test or Chi-square 2 × 2 contingency table (*p* < 0.05).

Eggs	Pupae Formation (Number of Pupae/Larvae)	Pupae Weight (mg)	Adult Emergence (Number of Adults/Larvae)	Fecundity (Number of Eggs/Day)
*S. littoralis*	23/23	10.6 ± 0.3 b	23/23 b	21.7 ± 4.9
*E. kuehniella*	20/23	9.4 ± 0.3 a	14/20 a	31.1 ± 2.6
Statistics	*χ*^2^ = 3.21; *p* = 0.07	*t* = 2.51; *p* = 0.02	*χ*^2^ = 11.19; *p* < 0.001	*t* = −1.62; *p* = 0.13

**Table 3:** The response time (minutes) of the first choice of *Chrysoperla carnea* L3 larvae under different conditions of predator number (one or two larvae) and feeding status (fed or fasted in the previous 24 h), analyzed by Student’s *t*-test (*p* < 0.05).

Feeding Status	Fasted		Fed	
Larvae Number	One Larva	Two Larvae	One Larva	Two Larvae
Egg	6.73 ± 1.21	4.03 ± 0.76	3.94 ± 0.87	3.42 ± 0.60
Larva	5.77 ± 1.49	4.00 ± 1.31	7.27 ^1^	2.85 ± 1.13
Statistics	*t* = 0.43; *p* = 0.67	*t* = 0.03; *p* = 0.98	-	*t* = 0.32; *p* = 0.75

^1^ Only one choice for *S. littoralis* larva; statistics could not be performed.

**Table 4:** The development of the preimaginal stages of *Chrysoperla carnea* after being fed different diets. The letters stand for significant differences between treatments by unifactorial ANOVA followed by LSD test or Chi-square 2 × 2 contingency tables (*p* < 0.05). Asterisks stand for controls in two by two analysis.

Diet ^1^	L3 Duration (Days)	Pupae Duration (Days)	Pupae Formation (Pupae/Larvae)	Pupae Weight (mg)	Adult Emergence (Adults/Larvae)
E	3.31 ± 0.10 a	9.65 ± 0.09 b	68/70 *	12.31 ± 0.31 c	65/70 *
ES	5.78 ± 0.28 b	10.83 ± 0.16 c	58/70	10.63 ± 0.27 b	41/70
EA	3.14 ± 0.05 a	8.82 ± 0.07 a	70/70 *	10.01 ± 0.22 ab	65/70 *
EAS	5.26 ± 0.13 b	9.69 ± 0.15 b	64/70	9.85 ± 0.28 a	44/70
Statistics	*F*_3,36_ = 71.12; *p* < 0.001	*F*_3,208_ = 56.42; *p* < 0.001	E/ES: *χ*^2^ = 7.94; *p* = 0.005 EA/EAS: *χ*^2^ = 6.27; *p* = 0.012	*F*_3,136_ = 16.66; *p* < 0.001	E/ES: *χ*^2^ = 22.38; *p* < 0.001 EA/EAS: *χ*^2^ = 22.38; *p* < 0.001

^1^ E: *Ephestia kuehniella* eggs until pupation; ES: *E. kuehniella* eggs until L3 larvae, and live *Spodoptera exigua* larvae until pupation; EA: *E. kuehniella* eggs and *Artemia* spp. cysts until pupation; EAS: *E. kuehniella* eggs and *Artemia* spp. cysts until L3 larvae, and live *S. exigua* larvae until pupation.

**Table 5:** The reproduction and survival of *Chrysoperla carnea* adults after been fed different diets. The letters stand for significant differences between treatments by unifactorial ANOVA followed by LSD test (*p* < 0.05).

Diet ^1^	Fecundity (Number of Eggs/Female and Day)	Fertility (% Neonate Larvae/Eggs)	Adult Survival (% Live Adults/Total Adults)
E	34.23 ± 1.61 c	81.67 ± 2.33	77.78 ± 8.24
EA	34.29 ± 1.00 c	76.36 ± 4.18	86.12 ± 6.68
ES	11.54 ± 0.92 a	85.43 ± 0.95	88.87 ± 3.52
EAS	17.07 ± 0.87 b	82.43 ± 1.99	80.57 ± 6.68
Statistics	*F*_3,52_ = 106.26; *p* < 0.001	*F*_3,20_ = 2.05; *p* = 0.14	*F*_3,20_ = 0.60; *p* = 0.62

^1^ E: *Ephestia kuehniella* eggs until pupation; EA: *E. kuehniella* eggs and *Artemia* spp. cysts until pupation; ES: *E. kuehniella* eggs until L3 larvae, and live *Spodoptera exigua* larvae until pupation; EAS: *E. kuehniella* eggs and *Artemia* spp. cysts until L3 larvae, and live *S. exigua* larvae until pupation.

## Data Availability

Data are available on personal request.
